# PINK1 suppresses alpha-synuclein-induced neuronal injury: a novel mechanism in protein phosphatase 2A activation

**DOI:** 10.18632/oncotarget.21554

**Published:** 2017-10-06

**Authors:** Weiwei Yang, Xue Wang, Jia Liu, Chunli Duan, Ge Gao, Lingling Lu, Shun Yu, Hui Yang

**Affiliations:** ^1^ Department of Neurobiology, Center for Parkinson’s Disease, Beijing Institute for Brain Disorders, Key Laboratory for Neurodegenerative Diseases of the Ministry of Education, Capital Medical University, Beijing, China; ^2^ Department of Neurobiology, Xuanwu Hospital, Capital Medical University, Beijing, China

**Keywords:** PINK1, alpha-synuclein (α-Syn), Parkinson’s disease (PD), protein phosphatase 2A (PP2A), Src kinase, Gerotarget

## Abstract

Alpha-synuclein (α-Syn) and phosphatase and tensin homolog deleted on chromosome ten (PTEN)-induced putative kinase (PINK) 1 are proteins found in Lewy bodies, which are a pathological hallmark of Parkinson’s disease (PD). PINK1 overexpression suppresses α-Syn-induced phenotypes and increases lifespan and health in an animal model of PD. It has been suggested that the two proteins regulate protein phosphatase (PP) 2A activity, but the underlying mechanisms and neuroprotective action of PP2A against PD-associated pathology are unknown. We found that α-Syn overexpression in SK-N-SH neuroblastoma cells and primary cortical neurons caused mitochondrial dysfunction and cell injury *via* phosphorylation of PP2A at Tyr307 and inhibition of its activity. Concomitant overexpression of PINK1 reversed this effect and restored the activity. The level of phospho-activated Src was increased in cells overexpressing α-Syn, which was reversed by co-expressing PINK1, suggesting that the latter suppressed α-Syn-induced PP2A inactivation by inhibiting Src activity. Calmodulin/Src complex formation was also enhanced in α-Syn-overexpressing cells, which was reversed by co-expression of PINK1 as a result of reduced mitochondrial Ca^2+^ releasing. Interestingly, the protective effects of PINK1 in α-Syn induced models were abolished by treatment with the PP2A inhibitor okadaic acid, indicating that PP2A is a target of PINK1. These findings indicate that PINK1 protects against α-Syn-induced neurodegeneration by promoting the dissociation of the calmodulin/Src complex and inhibiting Src, thereby enhancing PP2A activity. This was supported by the observation that PP2A activity was decreased in PD patients, which was negatively correlated with Hoehn and Yahr scores. Our results provide novel insight into the mechanisms underlying neurodegeneration in PD as well as possible avenues for therapeutic intervention in the treatment of this disease.

## INTRODUCTION

Parkinson’s disease (PD) is a neurodegenerative disease characterized by motor dysfunction—including tremor, muscle rigidity, and abnormal posture and pace—which is accompanied by a series of non-motor symptoms such as olfactory dysfunction, gastrointestinal disturbance, depression, and cognitive impairment [1]. PD results from the progressive degeneration and selective loss of dopaminergic neurons in the substantia nigra of the midbrain and cytoplasmic inclusions known as Lewy bodies that are formed in the remaining neurons [2].

The gene encoding α-synuclein (α-Syn)—the main component of Lewy bodies—was the first protein to be linked to PD pathogenesis; recent studies have suggested that posttranslational modification of α-Syn, especially phosphorylation at Ser129, plays a critical role in this process [3]. Under normal conditions, the level of α-Syn phosphorylated at Ser129 is very low ( < 4%); however, this is increased in Lewy bodies ( > 90%), indicating that α-Syn phosphorylation contributes to PD pathogenesis and progression [4, 5]. The phosphorylation of α-Syn is regulated by various kinases, including casein kinase [6], G-protein-coupled receptor kinase [7], and polo-like kinase [8]. Protein phosphatase (PP) 2A is the main α-Syn Ser/Thr phosphatase whose activity attenuates α-synucleinopathy [9].

Phosphatase and tensin homolog deleted on chromosome ten (PTEN)-induced putative kinase (PINK) 1—another component of Lewy bodies—is located in the cytosol and in mitochondria and is associated with PD. Previous studies have shown that PINK1 protected neurons against α-Syn-induced neurotoxicity in a *Drosophila* model of PD [10, 11]. PINK1 overexpression in dopaminergic neurons in *Drosophila* suppressed α-Syn-induced phenotypes [11] and increased lifespan as well as health [10]. Although the precise function of PINK1 is not well known, it has been shown to protect neurons from cellular stressors such as high levels of α-Syn by suppressing cytochrome c release and thereby inhibiting apoptosis [12, 13]. We previously demonstrated that loss of PINK1 function decreased PP2A activity in both cultured dopaminergic cells and the mouse striatum [14], suggesting that the protective activity of PINK1 is related to suppress PP2A inactivation.

PP2A is a major Ser/Thr phosphatase, which activity involves in regulating cellular metabolism. PP2A consists of scaffold subunit (A or PR65 subunit), regulatory subunit (B subunit), and catalytic subunit (C subunit). It was reported that PP2A was inactivated by phosphorylation at Tyr307 by upstream Src kinase activation [15, 16]. PP2A modulates various cellular processes including cell development and proliferation as well as intracellular signaling [17]. α-Syn overexpression was found to decrease PP2A activity, which promoted α-synucleinopathy and α-Syn-induced cytotoxicityin PD pathogenesis [18-20]. Additionally, Src tyrosine kinase catalyzes PP2A Tyr307 phosphorylation and suppresses its phosphatase activity, which may involve formation of a calmodulin-Src complex [21]. We previously showed that α-Syn overexpression inhibited PP2A by inducing phosphorylation of PP2A at Tyr307 *via* formation of a Src/calmodulin complex [22], indicating that PP2A is critical for α-Syn induced neurodegeneration. In addition, PINK1 overexpression protected cells against α-Syn induced cytotoxicity; however, it is unknown whether this effect involves PP2A activation.

In the present study, we demonstrated for the first time that PINK1 overexpression reverseed α-Syn-induced PP2A Tyr307 phosphorylation and inhibition of PP2A activity by preventing the formation of calmodulin-Src complexes, thereby attenuating mitochondrial dysfunction and neuronal apoptosis. Furthermore, the PP2A inhibitor okadaic acid (OA) blocked the neuroprotective effects of PINK1, whereas C2-ceramide-induced PP2A activation alleviated α-Syn-induced cell death. These results indicate that PP2A acts downstream of PINK1 to mediate neuroprotection in the presence of α-Syn, providing insight into the pathogenesis of PD and other neurodegenerative diseases.

## RESULTS

### PINK1 attenuates α-Syn-induced mitochondrial dysfunction and apoptosis

**Figure 1:**
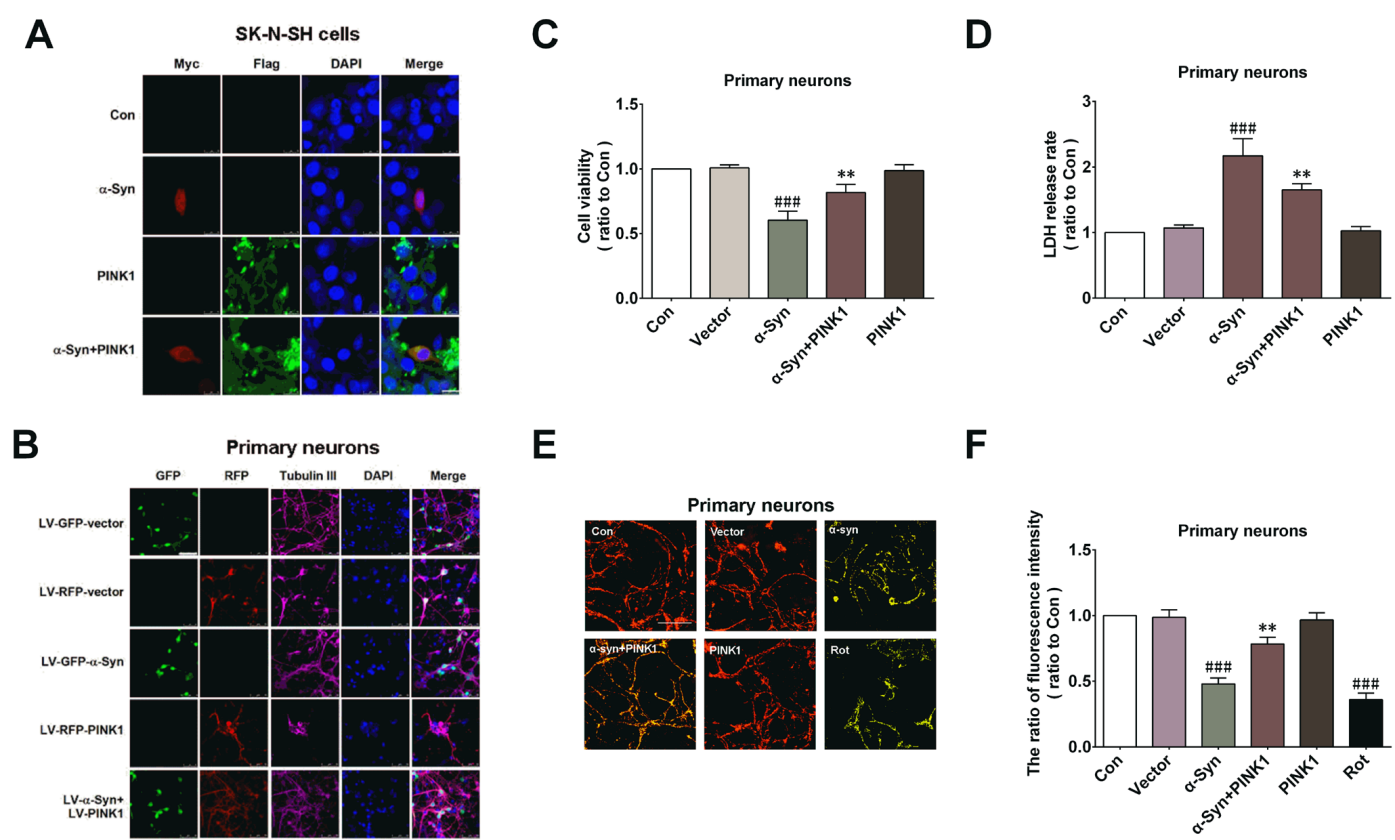
PINK1 attenuates α-Syn-induced mitochondrial dysfunction and cytotoxicity SK-N-SH cells were transfected with α-Syn/myc, PINK1/flag, or both vectors for 24 h. **A.** Cells were fixed 24 h later and labeled with antibody against myc or flag. Nuclei were counterstained with DAPI. **B.** Cells were transfected with myc, flag, myc/α-Syn, or flag/PINK1 vector or co-transfected with myc/α-Syn and flag/PINK1 vectors for 24 h. **C.**, **D.** Cell viability and cytotoxicity were measured with the MTT **C.** and LDH **D.** assays. **E.** MMP was detected with the JC-1 probe. **F.** Quantitative analysis of fluorescence intensity in JC-1-treated cells. Bar = 10 μM. Data are expressed as mean ± SD (*n* = 6). ^###^*P* < 0.001 *vs*. control (Con); **P < 0.01 *vs*. α-Syn (one-way analysis of variance).

We investigated whether PINK1 protected cells against injury induced by α-Syn overexpression. α-Syn and PINK1 were expressed individually or else co-expressed for 24h with plasmids or lentivirus (LV) in SK-N-SH neuroblastoma cells or primary cortical neurons; the overexpression of these proteins was confirmed by immunocytochemistry (Figure 1A, 1B). Cell viability and cytotoxicity were measured with the 3-(4,5-dimethylthiazol-2-yl)-2,5-diphenyltetrazolium bromide (MTT) and lactate dehydrogenase (LDH) assays. The results showed that PINK1 abrogated α-Syn overexpression-induced cell injury (Figure 1C, 1D). Mitochondrial dysfunction is a critical factor in PD pathogenesis; it has been reported that α-Syn overexpression resulted in mitochondrial dysfunction [23]. We therefore examined mitochondrial function by evaluating mitochondrial membrane potential (MMP). α-Syn overexpression markedly decreased MMP, similar to the positive control rotenone (100nM for 24h); this effect was abolished by co-expression of PINK1 (Figure 1E, 1F). Thus, PINK1 attenuated α-Syn overexpression-induced cell injury by improving mitochondrial function.

**Figure 2:**
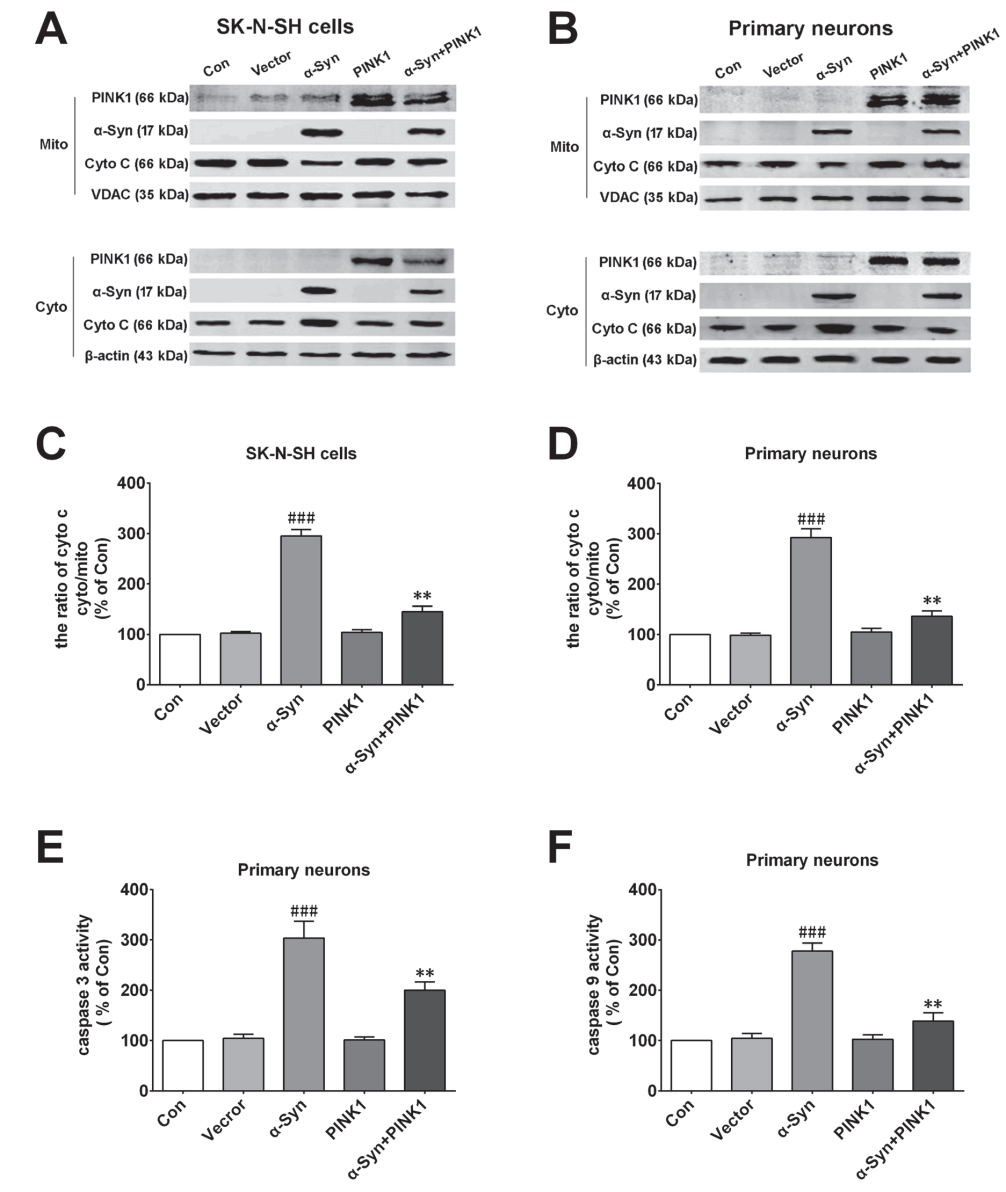
PINK1 protects cells against mitochondrial dependent apoptosis induced by α-Syn overexpression **A.**, **B.** Release of Cyto C from mitochondria into the cytoplasm was evaluated by western blotting in SK-N-SH cells (A) and rat primary cortical neurons (B). **C.**, **D.** Quantification of Cyto C in the cytoplasm and mitochondria of SK-N-SH cells (C) and rat primary cortical primary neurons (D). **E.**, **F.** Caspase-3 and -9 activities in rat primary cortical primary neurons. Data are expressed as mean ± SD (*n* = 6). ^###^*P* < 0.001 vs. control (Con); **P < 0.01 vs. α-Syn (one-way analysis of variance).

Previous studies have shown that α-Syn overexpression accelerated neurodegeneration by inducing apoptosis. To further investigate the effects of α-Syn and PINK1 on apoptosis, we measured the levels of cytochrome (Cyto) C released from mitochondria to the cytoplasm, which is a major step in the induction of mitochondrial apoptosis. α-Syn overexpression stimulated Cyto C release from mitochondria to the cytoplasm in SK-N-SH cells and primary neurons, which was inhibited in cells overexpressing both α-Syn and PINK1 (Figure 2A, 2B). We also examined the activation of caspase-9 and -3; the former was exclusively activated in mitochondrial apoptosis whereas the latter was activated in all apoptosis pathways. The results showed that PINK1 co-expression reversed α-Syn overexpression-induced caspase-9 and -3 activation (Figure 2C-2F). These findings indicated that PINK1 abrogated mitochondrial apoptosis induced by α-Syn overexpression.

### PINK1 reverses phospho-inhibition of PP2A caused by α-Syn overexpression

**Figure 3:**
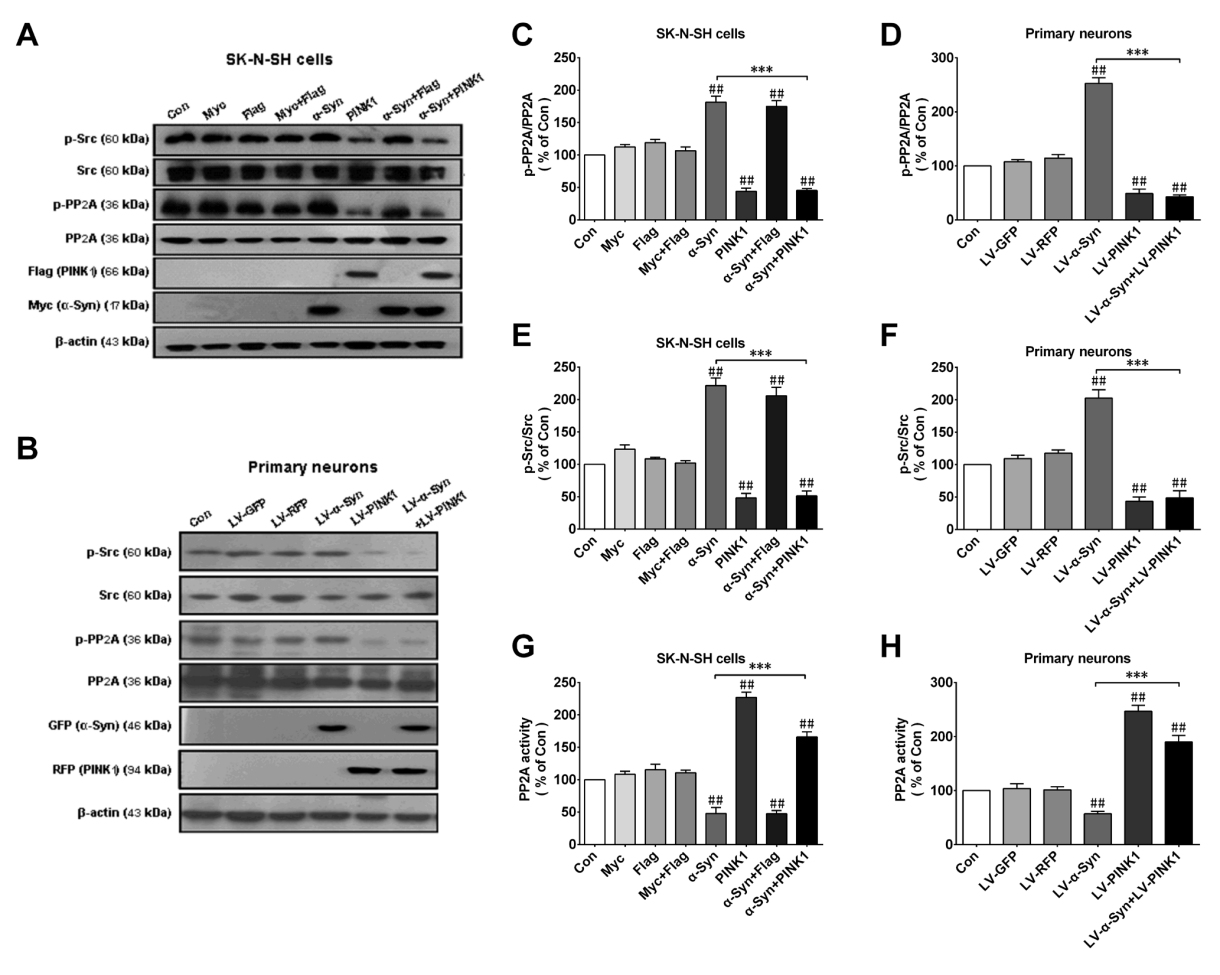
PINK1 overexpression reverses α-Syn-induced PP2A phospho-inhibition and Src phospho-activation SK-N-SH cells were transfected with α-Syn/myc, PINK1/flag, or both vectors for 24 h. **A.**, **B.** Expression of p-PP2A (Tyr307), total PP2A, p-Src (Tyr416), Src, myc/α-Syn, and flag/PINK1 was assessed by western blotting in SK-N-SH cells **A.** and rat primary cortical primary neurons **B.**, with β-actin used as the loading control. **C.**, **D.** Quantitative analysis of p-PP2A levels in SK-N-SH cells **C.** and rat primary cortical primary neurons **D.**. **E.**, **F.** Quantitative analysis of p-Src levels in SK-N-SH cells **E.** and rat primary cortical primary neurons **F.**. **G.**, **H.** PP2A activity was measured in SK-N-SH cells **G.** and rat primary cortical primary neurons **H.**. Data are expressed as mean ± SD (*n* = 6). ^##^*P* < 0.01 *vs*. control (Con); ***P < 0.001 *vs*. α-Syn (one-way analysis of variance).

It was previously reported that α-Syn aggregation reduced PP2A activity in the brains of patients with Lewy bodies. We have also shown that loss of PINK1 function decreased PP2A activity in a murine PD model, suggesting that α-Syn and PINK1 reciprocally regulated PP2A activity. To test this possibility, we measured the level of phosphorylated (p-)PP2A(Tyr307) by western blotting in SK-N-SH cells and primary cortical neurons. α-Syn and PINK1 overexpression enhanced and suppressed p-PP2A level, respectively, with complete reversal of the α-Syn-induced increase in p-PP2A expression in SK-N-SH cells co-expressing both proteins (Figure 3A-3D). Src tyrosine kinase acts upstream of PP2A and can catalyze PP2A Tyr307 phosphorylation; since Src activation is reflected by Tyr416 phosphorylation [24], we measured the level of p-Src by western blotting to assess Src activity. α-Syn overexpression increased p-Src levels, while co-expression of PINK1 reduced basal p-Src levels and reversed the increase in p-Src induced by α-Syn (Figure 3A, 3B, 3E, 3F). Furthermore, α-Syn overexpression inhibited PP2A activity, whereas PINK1 co-expression abrogated this effect (Figure 3G, 3H). These results indicated that PINK1 reversed α-Syn-induced PP2A inhibition *via* Src activation.

### PINK1 deficiency potentiates the α-Syn-induced increase in p-PP2A level

**Figure 4:**
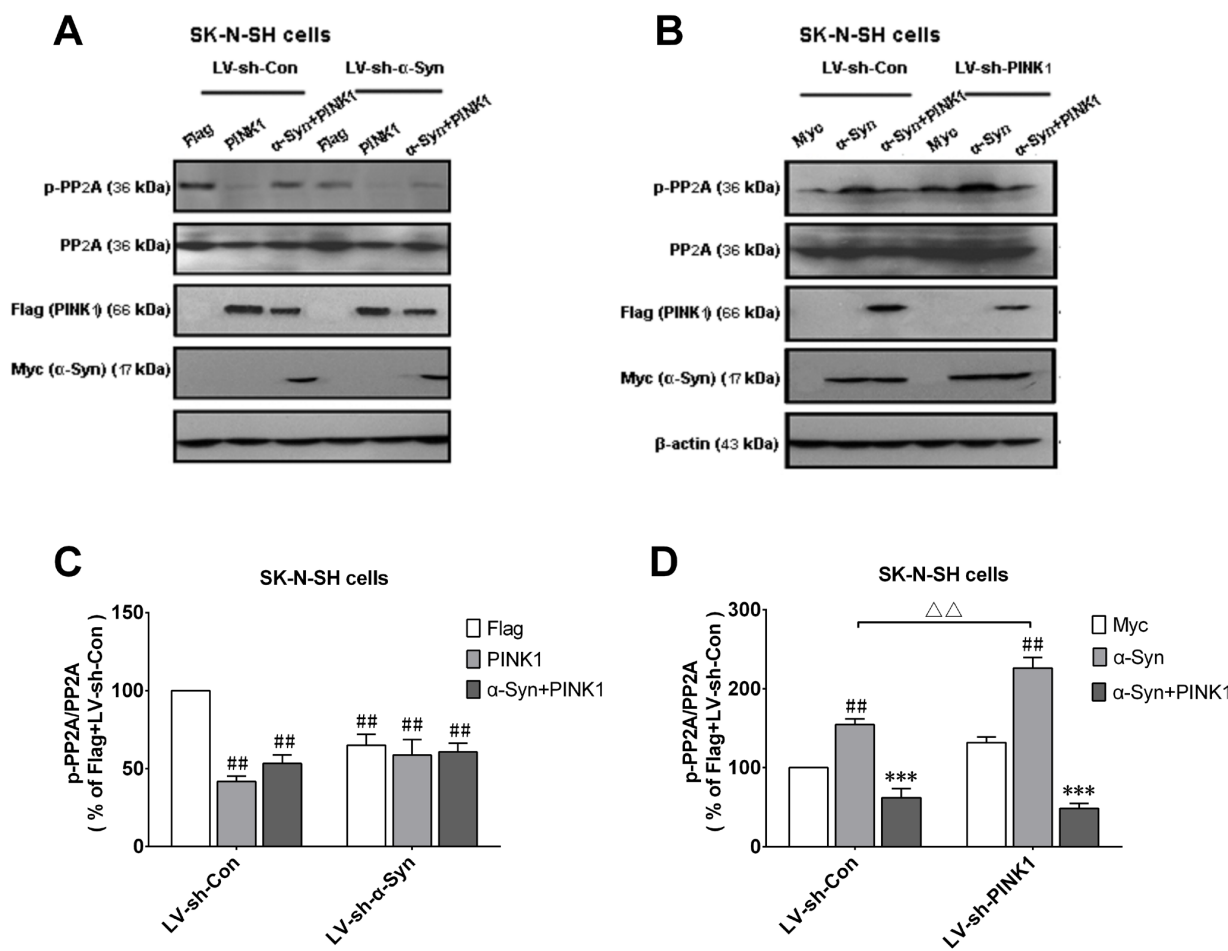
α-Syn-induced increase in p-PP2A is enhanced by PINK1 silencing SK-N-SH cells were infected with LV vectors encoding GFP-sh-PINK1, GFP-sh-α-Syn, or negative control shRNAs [LV-sh-con(PINK1) or LV-sh-con(α-Syn)] followed by transfection with indicated plasmids. **A.** Cells were infected with LV-sh-con (α-Syn) or LV-sh-α-Syn for 48 h, then transfected with flag or flag/PINK1 or co-transfected with α-Syn and PINK1 plasmids for 24 h; p-PP2A, PP2A, α-Syn/myc, and PINK1/flag expression was evaluated by western blotting. **B.** Cells were infected with LV-sh-con (PINK1) or LV-sh-PINK1 for 48 h, followed by transfection with myc or myc/α-Syn plasmids or co-transfection with α-Syn and PINK1 plasmids for 24 h. p-PP2A, PP2A, α-Syn/myc, and PINK1/flag expression was determined by western blotting. **C.** Quantitative analysis of p-PP2A level shown in **A.**. **D.** Quantitative analysis of p-PP2A level shown in **B.**. Data are expressed as mean ± SD (*n* = 6). ^##^*P* < 0.01 *vs*. Flag-LV-sh-Con; ***P < 0.001 *vs*. α-Syn; ^ΔΔ^*P* < 0.01 *vs*. α-Syn-LV-sh-Con (one-way analysis of variance).

To assess the effect of endogenous PINK1 on p-PP2A expression in the presence of high α-Syn levels, SK-N-SH cells were infected with LV vector encoding short hairpin (sh)RNA against α-Syn or PINK1. After 2 days, α-Syn knockdown caused a decrease in p-PP2A level, suggesting that endogenous α-Syn inhibits PP2A activity (Figure 4A, 4C). On the other hand, silencing PINK1 expression increased p-PP2A level relative to the control, which was further increased in cells overexpressing α-Syn (Figure 4B, 4D), implying that PINK1 deficiency caused PP2A phospho-inactivation. The latter effect was reversed by co-transfection of PINK1 and α-Syn (Figure 4B, 4D). Thus, PINK1 maintains PP2A activity by suppressing α-Syn-dependent phospho-inhibition of PP2A.

### PINK1 reverses α-Syn-induced binding of calmodulin to Src and Src-induced PP2A inhibition

**Figure 5:**
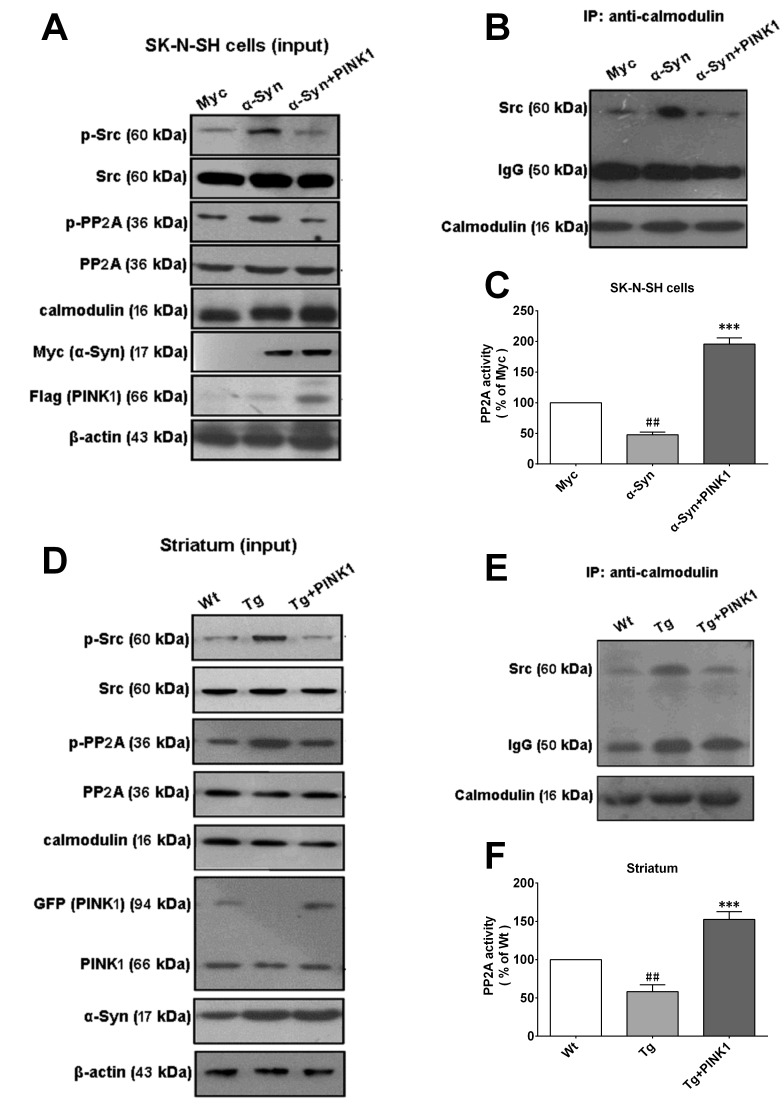
PINK1 overexpression reverses α-Syn-induced binding of calmodulin to Src **A.** SK-N-SH cells were transfected with vectors encoding myc/α-Syn or flag/PINK1 or both vectors for 24 h; p-Src and pPP2A levels were assessed by western blotting. **B.** Src immunoprecipitated using an anti-calmodulin antibody in cells overexpressing α-Syn with or without PINK1 co-expression. **C.** Changes in PP2A activity in cells overexpressing α-Syn. **D.** Western blot analysis of p-Src and p-PP2A levels in tissue lysates from Tg-α-Syn and WT mice with or without PINK1 overexpression in the striatum. **E.**Src immunoprecipitated from striatum tissue lysates of Tg-α-Syn and WT mice with or without PINK1 overexpression in the striatum. **F.** Changes in PP2A activity in the striatum of WT and Tg-α-Syn mice and Tg-α-Syn mice overexpressing PINK1. Data are expressed as mean ± SD(*n* = 6). ^##^*P* < 0.01 *vs*. Myc or WT; ***P < 0.001 *vs*. α-Syn or Tg (one-way analysis of variance).

Our previous works showed that PINK1 reversed α-Syn-induced PP2A inactivation by activating Src kinase; however, it is unclear how PINK1 and α-Syn regulate Src phosphorylation. Previous studies have shown that PINK1 deficiency stimulated mitochondrial permeability transition pore opening and increased cytosolic Ca^2+^ [25]. Moreover, α-Syn overexpression was shown to increase Ca^2+^ in H_2_O_2_-treated cells and in a mouse model of synucleinopathy [26, 27]. A Ca^2+^-calmodulin complex is formed when intracellular free Ca^2+^ is high; this activates downstream signaling cascades, including those involving phosphatidylinositol 3-kinase and Src kinase [21]. To determine whether reciprocal regulation of PP2A activity by PINK1 and α-Syn is mediated by Ca^2+^-calmodulin-dependent Src activation, we examined the effects of PINK1 and α-Syn on p-PP2A and p-Src levels (Figure 5A).

We speculated that PINK1 blocked α-Syn-dependent interactions between calmodulin and Src. To test this hypothesis, we immunoprecipitated SK-N-SH cell lysates using an anti-calmodulin antibody. A larger amount of Src was precipitated from lysates of cells overexpressing α-Syn as compared to PINK1/α-Syn (Figure 5B). Moreover, α-Syn overexpression decreased PP2A activity, an effect that was reversed by PINK1 co-expression (Figure 5C). These results indicated that overexpressing α-Syn leaded to the formation of calmodulin-Src complexes, a process that is disrupted by PINK1.

To evaluate this effect *in vivo*, p-PP2A and p-Src levels were measured in the striatum of transgenic (Tg)-α-Syn and wild-type (WT) mice by western blotting. The results showed that PINK1 reversed the α-Syn-induced increase in p-PP2A and p-Src levels(Figure 5D). To examine the formation of the calmodulin-Src complex, immunoprecipitation (IP) was performed using lysates from the striatum of Tg-α-Syn and WT mice. Consistent with the above results, a greater amount of Src was precipitated with calmodulin from Tg-α-Syn as compared to WT tissue lysates (Figure 5E); complex formation was abolished by overexpressing PINK1 in the striatum. Moreover, PP2A activity was decreased in Tg-α-Syn mice, but was restored when PINK1 was co-expressed (Figure 5F). Therefore, high level of α-Syn leads to formation of calmodulin-Src complexes and Src activation and enhances phospho-inhibition of PP2A; on the other hand, complex formation is disrupted in the presence of PINK1, which increases PP2A activity.

### PINK1 buffers α-Syn-induced [Ca^2+^] elevation

**Figure 6:**
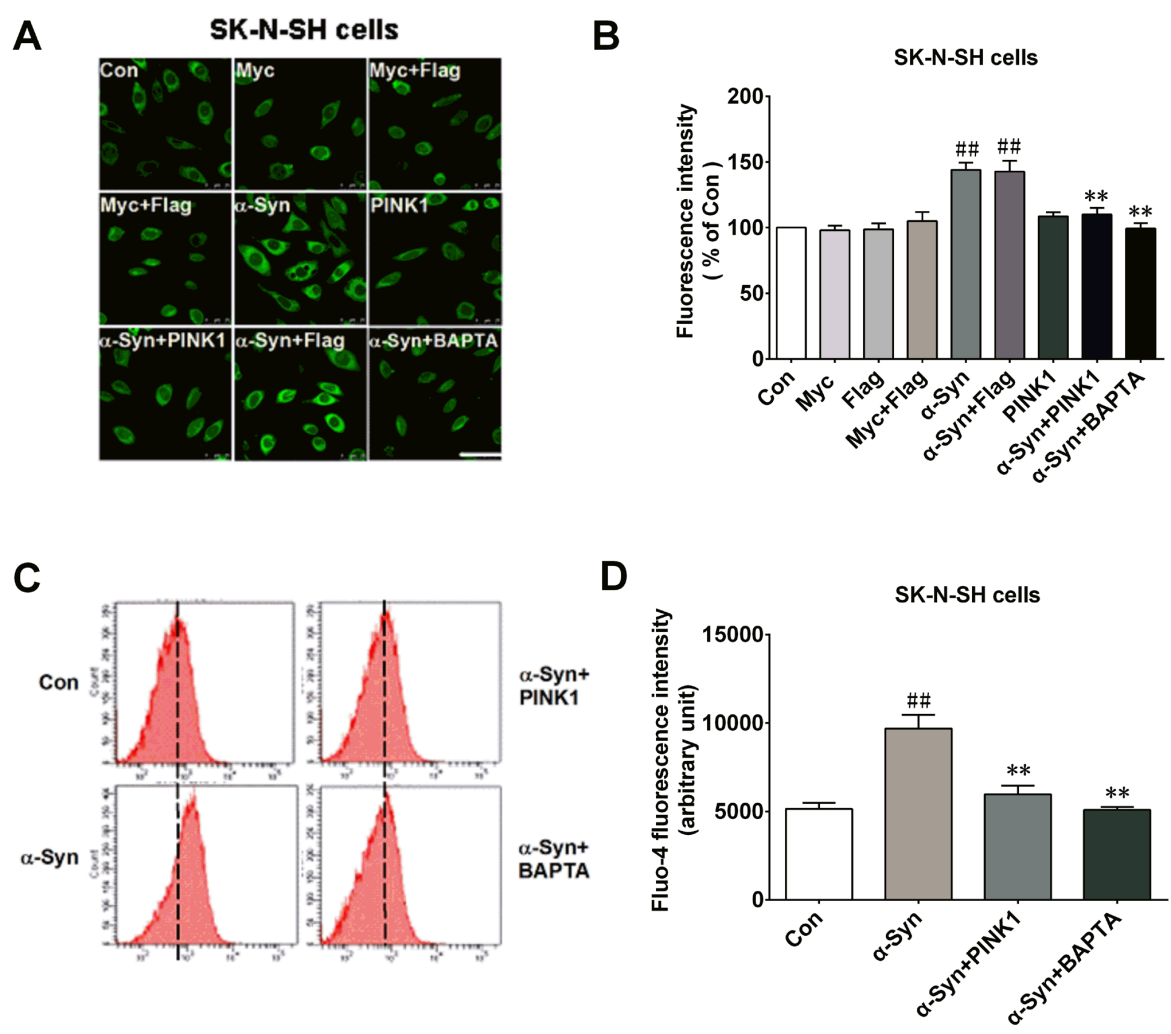
PINK1 buffers the increase in [Ca^2+^] induced by α-Syn overexpression SK-N-SH cells were transfected with indicated plasmids for 24 h with or without BAPTA/AM (50 μM for 1 h). **A.** Cytosolic [Ca^2+^] was measured by analyzing mean fluorescence intensity in each group by confocal microscopy. Bar = 25 μM. **B.** Fluorescence intensity determined from confocal micrographs. **C.** Fluo-4 fluorescence intensity wasincreased in α-Syn-overexpressing cells, which was abolished by treatment with BAPTA/AM (50 μM for 1 h; α-Syn+BAPTA/AM group) or co-transfection of PINK1 (α-Syn+PINK1 group), as determined by flow cytometry. **D.** Cytosolic [Ca^2+^] measured by calculating mean Fluo-4 fluorescence intensity in each group. Data are expressed as mean ± SD (*n* = 6). ^##^*P* < 0.01 *vs*. control (Con); **P < 0.01 *vs*. α-Syn(one-way analysis of variance).

Calmodulin is activated by increased cytosolic Ca^2+^; previous studies have reported an increase in Ca^2+^ in SK-N-SH cells and in a mouse model of synucleinopathy upon α-Syn overexpression [22, 26]. We investigated whether PINK1 can reverse the elevation in Ca^2+^ induced by α-Syn overexpression. Cytosolic Ca^2+^ levels were higher in α-Syn-overexpressing cells than in control cells after 24 h, an effect that was reversed by co-expressing PINK1 or by treatment with the cell-permeable Ca^2+^ chelator BAPTA/AM (50 μM, 60 min), as determined by confocal microscopy (Figure 6A, 6B) and flow cytometry (Figure 6C, 6D).

### PINK1 fails to protect against cellular injury induced by α-Syn when PP2A is inactivated

**Figure 7:**
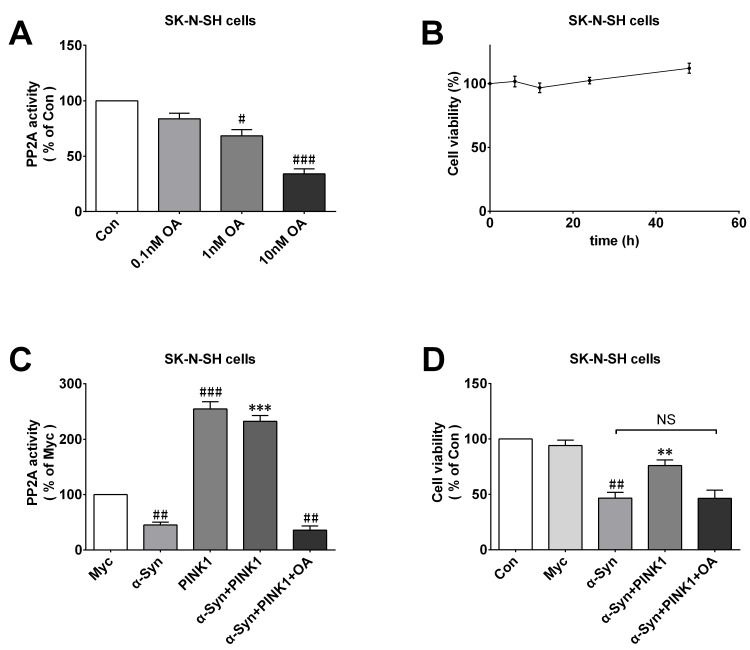
PINK1 abolishes the protective effects against α-Syn-induced cell injury in PP2A inactive condition **A.** OA decreased PP2A activity in dose-dependent manner. **B.** Viability of SK-N-SH cells treated with 10 nM OA for 0, 6, 12, 24, or 48 h, as determined with the MTT assay. **C.**, **D.** Changes in PP2A activity **C.** and cell viability **D.** in Con, myc, α-Syn, PINK1,α-Syn+PINK1, and α-Syn+PINK1+OA groups. Data are expressed as mean ± SD (*n* = 6). ^#^*P* < 0.05, ^##^*P* < 0.01, ^###^*P* < 0.001 *vs*. control (Con) or Myc; **P < 0.01, ***P < 0.001 *vs*. α-Syn, NS, not significant (one-way analysis of variance).

We examined whether PP2A activity involved the neuroprotective effect of PINK1 against α-Syn-induced cell injury. SK-N-SH cells were treated with different concentrations of the PP2A inhibitor OA (0, 0.1, 1, or 10 nM for 12 h) to suppress PP2A activity. We found that 10nM OA was most effective in inhibiting PP2A activity (Figure 7A) and had no effect on cell viability (Figure 7B); this concentration was therefore selected for follow-on experiments. To investigate the effect of OA on cells overexpressing or co-expressing α-Syn and PINK1, we measured P22A activity in each group. The results showed that although PINK1 reversed α-Syn-induced PP2A inactivation, OA treatment completely abolished this effect (Figure 7C), indicating that PINK1 mediated neuroprotection by maintaining PP2A activity. Additionally, the results of the MTT assay showed that OA abrogated the protective effect of PINK1 on cell viability (Figure 7D). These results suggested that the increased in PP2A activity induced by PINK1 overexpression protects neurons against the toxic effects of α-Syn.

### PP2A activity is reduced in PD patients and is negatively correlated with Hoehn and Yahr (H&Y) score

H&Y score, a widely used clinical rating scale, is used to describe the symptom progression of PD. This scale includes stages 1 through 5, correlated with motor deficits, deterioration in quality of life, and dopaminergic loss. The demographic information of 50 PD patients and 50 healthy control subjects included in this study is shown in Table 1. Mean ages for the PD and control groups were 57.8 years (range: 30-78 years) and 62.3 years (range: 32-86 years), respectively. The male-to-female ratios were similar between the two groups.

**Table 1:** Demographic information of study subjects.

	Controls (n = 50)	Patients (n = 50)	P
Age (years)	62.3 ± 10.1	57.8 ± 10.7	n.s.
Sex (male:female)	41:9	37:13	n.s.
H&Y scores		2.7 ± 0.81	

H&Y, Hoehn and Yahr; n.s., not significant.

Our finding that PINK1 protects against α-Syn-induced cell injury by enhancing PP2A activity implied that PP2A plays a key role in PD pathogenesis. We therefore examined PP2A activity in the plasma of PD patients and control subjects with the PP2A activity assay and by co-IP. The level of p-PP2A/C was increased (Figure 7A) whereas PP2A activity was decreased (Figure 7B, 7C) in PD patients as compared to controls. We also found that PP2A activity was negatively correlated with H&Y scores (r^2^ = 0.487) (Figure 7D), suggesting that PP2A is involved in the pathogenesis of PD.

## DISCUSSION

PD is a neurodegenerative disease that is common among the elderly. Various factors are involved in PD pathogenesis, with genetics playing a key role [28]. α-Syn and PINK1 are two proteins related to PD; previous studies have shown that PINK1 protected against α-Syn induced cell injury by unknown mechanisms [10-12]. The present study found that PINK1 prevents α-Syn-induced cell death by suppressing Src activity with concomitant PP2A activation, possibly *via* inhibition of mitochondrial Ca^2+^ release (Figure 9).

**Figure 8:**
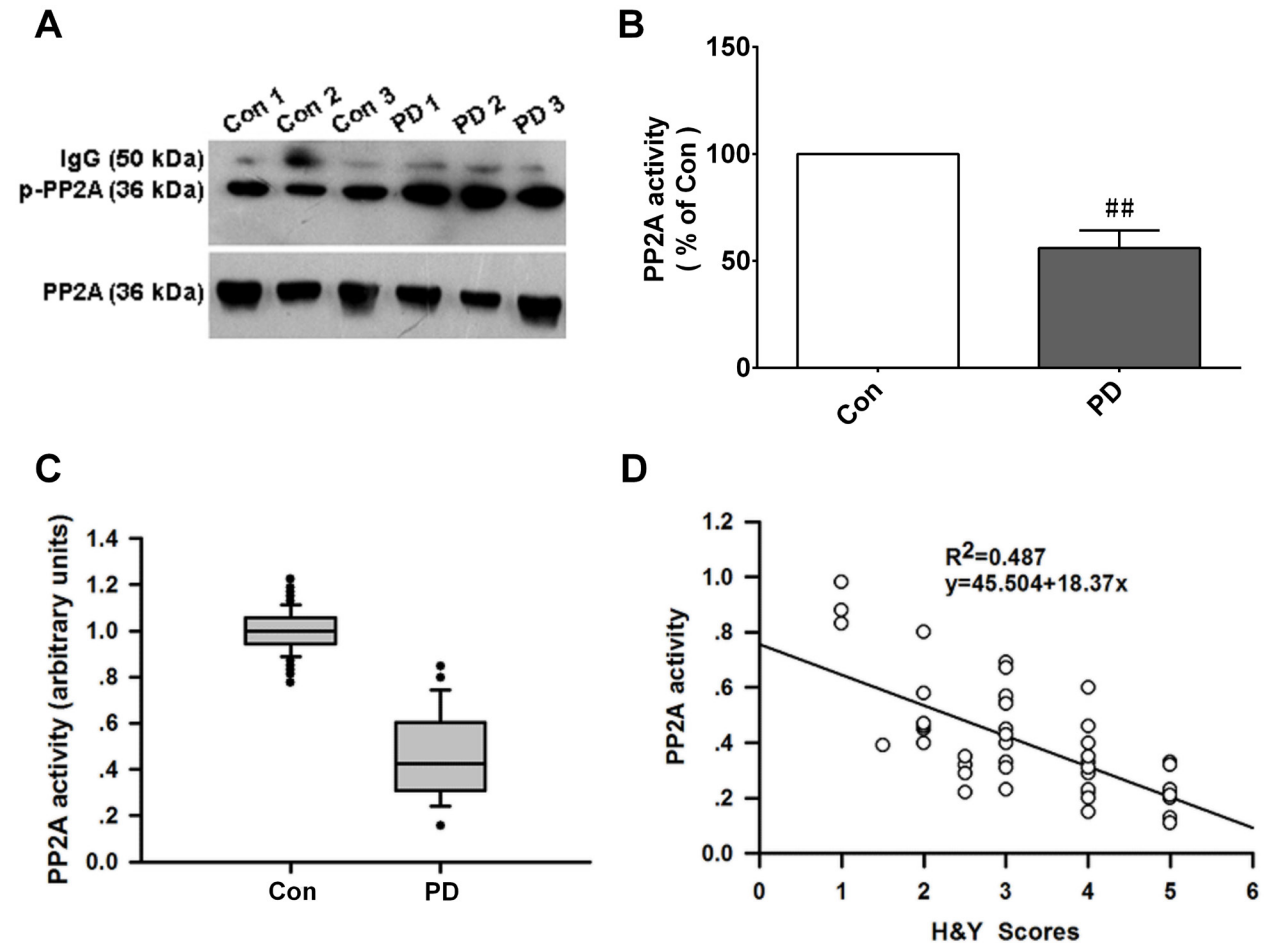
PP2A activity is decreased in PD patients and is negatively correlated with H&Y scores **A.** PP2A activity in plasma was detected by IP. p-PP2A level was elevated in the plasma of PD patients as compared to control subjects. **B.** Changes in PP2A activity in PD *vs*. control (Con) group. Data are expressed as mean ± SD (*n* = 50, two independent experiments). ^##^*P* < 0.01 (t test). **C.** PP2A activity was decreased in the PD relative to the CTL group (Mann-Whitney test). **D.** Pearson correlation analysis showing a negative correlation between PP2A activity level and H&Y scores (Pearson correlation analysis, r^2^ = 0.487; *n* = 50).

**Figure 9:**
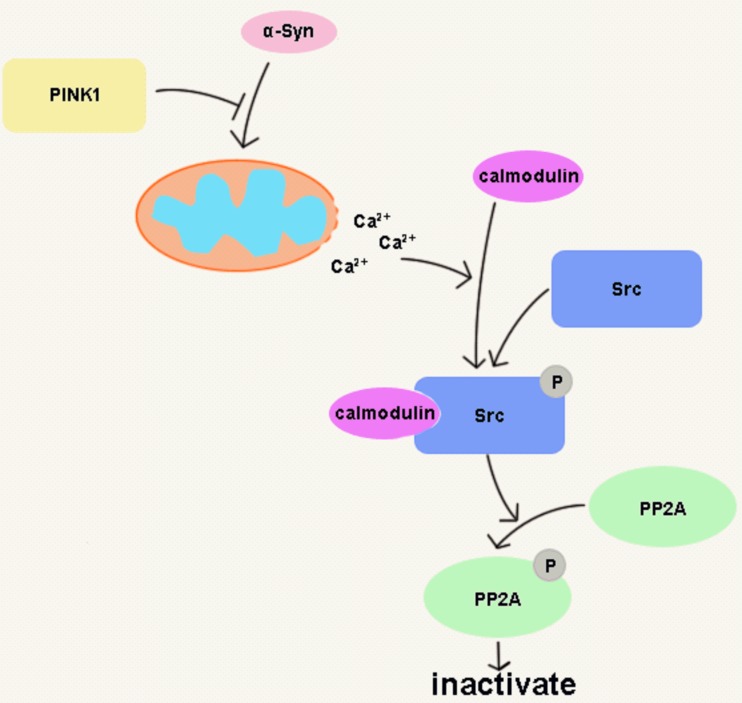
PINK1 prevents α-Syn overexpression-induced cell death by PP2A activation PINK1 blocked α-Syn induced Ca^2+^ release from mitochondria to cytoplasm and consequent calmodulin-Src complex formation, thereby suppressing Src activation and enhancing PP2A activity.

α-Syn is the main component of Lewy bodies; its overexpression has been shown to be neurotoxic in a variety of cellular and animal models of PD. Furthermore, a functional link has been reported between α-Syn and PP2A, although the conclusions are controversial [19, 29, 30]. One study found that α-Syn overexpression activated PP2A, while phosphorylation of α-Syn Ser-129 by Polo-like-kinase 2 reduced PP2A activation induced by α-Syn *in vitro* [29]. Similarly, others have reported that interaction with α-Syn leads to PP2A activation [31]. However, it was recently reported that Lewy body-like aggregations of α-Syn inhibited PP2A activity *in vitro* and *in vivo* [32]. In aging monkey brains, increased α-Syn phosphorylation and oligomerization were associated with lower PP2A activity [19]. We found here that high levels of α-Syn suppressed PP2A activity by activating Src kinase. The fact that α-Syn induces cell injury by PP2A inactivation suggests that the latter could be a therapeutic target to prevent α-Syn induced cytotoxicity. PINK1, another PD-related protein, protected cells against apoptosis induced by α-Syn [12, 13, 33], and PINK1 deficiency exacerbated α-Syn-induced neurodegeneration [34]. Our findings suggest that PINK1 inhibits mitochondrial Ca^2+^ release induced by α-Syn overexpression and prevents mitochondrial dysfunction and apoptosis by activating Src kinase *via* formation of calmodulin-Src complex, and thereby enhancing PP2A activity.

PINK1 is localized in the cytosol and mitochondria and plays an indispensable role in mitochondrial quality control [35]. Mutations of PINK1 have been identified in familial and sporadic PD cases [36]; moreover, PINK1 and α-Syn immunoreactivity were colocalized in a fraction (5%-10%) of neuronal inclusions resembling Lewy bodies in the brains of PD patients [37]. Our previous study demonstrated that PINK1 dysfunction impaired PP2A activity in cultured dopaminergic neurons and in a mouse PD model [14]. Here we showed that PINK1 overexpression suppressed mitochondrial Ca^2+^ release and enhanced PP2A activity even in the presence of α-Syn, thereby increasing cell viability by improving mitochondrial function. Moreover, PINK1 silencing in SK-N-SH cells overexpressing α-Syn reduced PP2A activity. These results indicate that PINK1 reverses α-Syn-induced mitochondrial and cell injury by targeting PP2A activity.

PP2A plays an important role in regulating cellular metabolism. PP2A dephosphorylates α-Syn at Ser129 and the resultant in activation of α-Syn exacerbates α-synucleinopathy [9]. Our work demonstrates that α-Syn impairs PP2A activity; inactivated PP2A leads to α-Syn phosphorylation and neurodegeneration in a positive feedback loop that accelerates disease progression. However, PINK1 disrupts this cycle and prevents neurodegeneration by enhancing PP2A activity. We also find that PP2A activity is decreased in PD patients as compared to normal subjects and is negatively correlated with H&Y scores; these results suggest that PP2A activity is closely related to the pathogenesis of PD and can serve as a diagnostic or therapeutic target.

In summary, PINK1 overexpression blocked α-Syn-induced mitochondrial Ca^2+^ release and reversed the binding of calmodulin to Src, leading to a decrease in Src activity and suppressing Src-mediated phospho-inhibition of PP2A. In addition, PP2A activation protected cells against α-Syn induced injury. Thus, PINK1 overexpression triggers several responses that protect against α-Syn-induced neurotoxicity. These findings provide evidence of a critical role for PP2A in PINK1-mediated neuroprotection against α-Syn and suggest that PINK1, PP2A, and Src as well as α-Syn are potential therapeutic targets for PD treatment.

## MATERIALS AND METHODS

### Plasmids and LV

Human WT-PINK1 and WT-α-Syn cDNA were obtained by reverse transcription PCR from human brain RNA using primers 5’-GAG TAT GGA GCA GTC ACT TAC AG-3’ and 5’-CAG CAC ATC AGG GTA GTC G-3’ for PINK1 (GenBank accession no. NM_032409.2) and 5’-CTG GAA GAT ATG CCT GTG GAT C-3’ and 5’-AGC ACT TGT ACA GGA TGG AAC-3’ for α-Syn (GenBank accession no. NM_000345.3). The cDNAs were directionally cloned into the pcDNA3.1-3×flag (for PINK1) and pcDNA3.1-myc (for α-Syn) plasmids (both from Invitrogen, Carlsbad, CA, USA); their orientations were verified by DNA sequencing. LV vectors encoding green fluorescent protein (GFP)-sh-α-Syn, negative control RNA [LV-sh-con (α-Syn)], GFP-sh-PINK1, negative control RNA [LV-sh-con (PINK1)], GFP-WT-α-Syn, GFP-WT-PINK1, and RFP-WT-PINK1 were generated by Genechem (Shanghai, China).

### Cell culture

Human SK-N-SH cells obtained from the American Type Culture Collection (Manassas, VA, USA) were grown in Dulbecco’s Modified Eagle’s Medium supplemented with 10% fetal bovine serum and transfected with plasmids using Lipofectamine 2000 (Invitrogen) according to the manufacturer’s instructions. Briefly, cells were seeded at 1 × 10^7^ cells per flask 1 day before transfection. The plasmids pcDNA3.1-myc (myc), pcDNA3.1-myc-α-Syn (α-Syn/myc), pcDNA3.1-flag (flag), or pcDNA3.1-flag-PINK1 (PINK1/flag) (9 μg) were mixed with 20 μl Lipofectamine 2000 in Opti-MEM (Invitrogen). Cells were incubated at 37°C in a CO_2_ incubator, and protein expression was verified by western blotting. In a separate experiment, SK-N-SH cells were infected with an LV vector encoding control shRNAs (sh-con α-Syn or sh-con PINK1), sh-α-Syn, or sh-PINK1; 3 days later, the cells were transfected with myc, flag, α-Syn/myc, or PINK1/flag plasmids for 24 h and then analyzed by western blotting.

### Primary cortical neuron culture

All experiments involving animals were approved by the Institutional Animal Care and Use Committee of Capital Medical University of Science and Technology (approval no. SCXK-2011-004) and were carried out in strict accordance with the National Institutes of Health Guide for the Care and Use of Laboratory Animals. Surgeries were performed under sodium pentobarbital anesthesia. Primary cortical neurons were prepared from C57/B6 mouse E14.5-E15.5 embryos and cultured in 3.5-cm dishes (1.4 × 10^6^ cells/dish) on cover slips coated with 100 μg/ml poly-l-lysine in Neurobasal medium (Invitrogen) supplemented with basic fibroblast growth factor (10 ng/ml), nerve growth factor (10 ng/ml), l-glutamine (0.5 mM), and B27 supplement without OA (1×). After 7 days, primary neurons were infected with LV gene transfer vectors.

### Transgenic mice

Male transgenic mice (18-22 g) overexpressing human α-Syn (Tg-α-Syn) under the control of the platelet-derived growth factor promoter were provided by Dr. Changan Jiang (Sichuan University, China) and maintained on a C57BL/C3H background. Transgenic mice and WT littermates were housed under a 12:12-h light/dark cycle at 20°C-23°C with free access to food and water.

### Generation of PINK1-overexpressing mice by LV infection

Male Tg-α-Syn mice (7 weeks old, 18-20 g) were anesthetized with 6% chloral hydrate (6 ml/kg by intraperitoneal injection). Stereotactic coordinates for injection into the striatum were as follows: bregma anteroposterior +1 mm; lateral ±1.8 mm; and ventral −2.7 mm from the dura, with the tooth bar set at 0 mm. GFP-encoding LV vector (106 TU) resuspended in a 2 μl-volume was injected (0.2 μl/min) into the striatum, with the needle left in place for an additional 15 min before slow withdrawal. The skin was sutured and mice were allowed to recover.

### MTT and LDH assays

To evaluate cell viability, 1 × 10^4^ SK-N-SH cells transfected with myc, α-Syn/myc, PINK1/flag, or α-Syn+PINK1 for 6, 12, 24, 48, or 72 h were seeded in 96-well plates. Some cultures were treated with the PP2A inhibitor OA (10 nM). The medium was replaced with MTT (Promega, Madison, WI, USA) at a final concentration of 0.5mg/ml followed by incubation for 4h. Cells were washed three times with phosphate-buffered saline (PBS); formazan crystals were dissolved in 100 μl dimethyl sulfoxide, and absorbance was read at 490nm microplate reader (PerkinElmer, Waltham, MA, USA). The LDH assay was performed using a kit (Roche Diagnostics, Mannheim, Germany) according to the manufacturer’s instructions. A 100μl aliquot of each supernatant was used to measure LDH release, with 100μl of preservation solution used as a blank to correct the optical density reading at 490nm. Each concentration was tested in triplicate, and the half-maximal effective concentration was determined as the average from five experiments.

### JC-1 staining for determination of MMP

MMP was measured using JC-1 (T4069; Sigma-Aldrich, St. Louis, MO, USA), a dual-emission membrane potential-sensitive probe that exists as a green fluorescent monomer at low MMP, according to the manufacturer’s instructions and as previously reported [38], but has red/orange fluorescence in the J-aggregate form at higher MMP. JC-1 (1.3 μg/ml) was added to cells cultured in 24-well plates after washing twice with PBS for 30 min at 37°C. The change in fluorescence at 488/530 nm (green) and 549/595 nm (red) was monitored by high-content screening, and the ratio of green/red fluorescence intensity was determined.

### Isolation of mitochondrial and cytoplasmic fractions

Mitochondria were isolated from transfected HEK293T cells or primary neurons using the Mitochondria/Cytosol Fractionation kit (Applygen Technologies, Beijing, China; C1260) as previously reported [39].

### Caspase-3 and -9 activity assays

Caspase-3 and -9 activities were measured with Caspase-3 and -9 colorimetric assay kits (Applygen; C1113 and C1119, respectively). The assay is based on spectrophotometric detection of the chromophore p-nitroanilide (pNA) after its cleavage from the labeled substrate LEHD-pNA. After transfection for 48 h, cells were centrifuged at 1,000 × *g* for 5 min. A 60μl volume of cell lysis buffer was added and the mixture was vortexed and incubated on ice for 10 min, followed by vortexing and centrifugation at 12,000 × *g* for 10 min at 4°C. Proteins were transferred to another 1.5ml centrifuge tube and the protein concentration was determined using a Bradford assay kit (GenMed Scientifics, Shanghai, China;GMS 30030.1). A 50μl volume of protein was added to the 96-well plate, and45 μl reaction buffer was added to each sample; 5 μl of 2 mM LEHD-pNA (100 μM final concentration) were then added, and the plate was sealed with Parafilm and incubated at 37°C for 2 h. The absorbance of samples was read at 405 nm with a spectrophotometer. One unit was defined as the amount of enzyme that cleaved 1.0 nmol of the colorimetric pNA-substrate per hour at 37°C under saturated substrate concentrations.

### Western blot analysis

Primary neurons from C57/B6 mice were infected with LV-GFP, LV-GFP-α-Syn, LV-RFP, or LV-RFP-PINK1, or co-infected with LV-GFP-α-Syn and LV-RFP-PINK1 for 3 days. The cells were harvested and lysed in TNE buffer (50 mM Tris-HCl [pH 7.4], 150 mM NaCl, and 1 mM EDTA; all from Sigma-Aldrich) containing 1% Nonidet (N)P-40 (Calbiochem, Billerica, MA, USA) and protease and phosphatase inhibitor cocktails (Roche Diagnostics, Indianapolis, IN, USA). Alternatively, cultured SK-N-SH cells were transfected with PINK1, α-Syn, or control plasmids for 24 h. Total cell extracts were centrifuged at 12,000 × *g* for 30 min, and the protein concentration in the supernatant was determined using a bicinchoninic acid (BCA) protein assay kit (Pierce, Rockford, IL, USA). For western blot analysis, protein lysates were separated by 4%-12% Bis-Tris sodium dodecyl sulfate-polyacrylamide gel electrophoresis (SDS-PAGE) (20 μg/lane) and transferred to polyvinylidene difluoride membranes that were probed with antibodies against myc, p-Src, cytochrome C, and β-actin (all from Sigma-Aldrich); flag (Proteintech, Chicago, IL, USA); Src (Novus Biologicals, Littleton, CO, USA); PP2A, p-PP2A, calmodulin, and α-Syn (all from Cell SignalingTechnologies, Danvers, MA, USA); and PINK1 (Abcam, Cambridge, MA, USA). β-Actin served as a loading control. This was followed by incubation with horseradish peroxidase-conjugated secondary antibodies (Santa Cruz Biotechnology, Santa Cruz, CA, USA). Protein bands were visualized by enhanced chemiluminescence and densitometry was performed using the Versadoc XL imaging system (Bio-Rad, Hercules, CA, USA).

### Immunocytochemistry and confocal microscopy

For immunocytochemistry, primary cortical neurons were seeded on poly-l-lysine-coated cover slips placed in Petri dishes and infected with LV-GFP, LV-GFP-α-Syn, LV-RFP, or LV-RFP-PINK1, or co-infected with LV-GFP-α-Syn and LV-RFP-PINK1 for 3 days. Neurons were then fixed with 3.7% paraformaldehyde for 20 min, washed in PBS, and permeabilized with 0.2% Triton X-100 in PBS for 10 min at room temperature. Fixed cells were incubated with an anti-tubulin III antibody (Abcam) overnight at 4°C, followed by incubation with Alexa Fluor 647-labeled secondary antibody for 1 h at room temperature. Cell nuclei were visualized by counterstaining with 4’,6-diamidino-2-phenylindole (DAPI; Invitrogen). Additionally, cultured SK-N-SH cells were transfected with myc/α-Syn or flag/PINK1, or co-transfected with myc/α-Syn and flag/PINK1 plasmids for 24 h. Cells were then fixed with 3.7% paraformaldehyde for 20 min, washed in PBS, and permeabilized with 0.2% Triton X-100 in PBS for 10 min at room temperature. After overnight incubation at 4°C with an anti-myc (Clontech, Mountain View, CA, USA) or anti-flag (ProteinTech) antibody, cells were labeled with Alexa Fluor 488- or 594-conjugated secondary antibody (1:500) for 1 h at room temperature. Nuclei were visualized by counterstaining with DAPI.

### IP analysis

The striatum was dissected from Tg-α-Syn or WT mice and lysed in TNE buffer containing 1% NP-40 and protease and phosphatase inhibitors. Homogenates were centrifuged at 12,000 × *g* for 30 min at 4°C. Protein concentration was determined using the BCA kit. Transfected SK-N-SH cell extracts (100 μg) or tissue extracts from Tg-α-Syn or WT mice (100 μg) were incubated overnight at 4°C with an anti-calmodulin antibody (3 μg; ProteinTech) with constant rotation. Protein G-sepharose beads (30 μl/tube) were washed three times (15 min/wash) in IP buffer (10 mM Tris-Cl [pH 7.5], 150 mM NaCl, 2 mM EDTA, and 0.5% Triton X-100) and incubated in the protein/antibody mixture with constant rotation at 4°C for 1 h. Beads were collected by centrifugation at 10,000 × *g* for 1 min and washed three times with IP buffer to remove non-specifically bound proteins. Washed beads were resuspended in SDS-PAGE loading buffer (60 μl/tube) and heated at 95°C for 5 min, then removed by centrifugation at 10,000 × *g* for 1 min. The supernatant was analyzed by western blotting.

### PP2A activity assay

Cultured SK-N-SH cells transfected with α-Syn and/or PINK1 plasmids for 24 h and then treated with OA (0, 0.1, 1, or 10 nM for 12 h) or vehicle were harvested and lysed. PP2A activity was detected using the PP2A Activity Assay kit (GenMed, Minneapolis, MN, USA) according to the manufacturer’s instructions and as previously reported [22]. Activity was determined by measuring the absorbance at 660 nm on a spectrophotometer (Bio-Rad).

### Cytosolic Ca^2+^ measurements

To estimate changes in cytosolic Ca^2+^, SK-N-SH cells were loaded with the Ca^2+^-sensitive fluorescent dye Fluo-4 (Dojindo Laboratories, Kumamoto, Japan) and florescence intensity (correlated with Ca^2+^) was analyzed by flow cytometry or confocal microscopy. Cells were transferred to microcentrifuge tubes 24 h after transfection with or without BAPTA/AM (50 μM for 1 h), then washed three times in Hank’s Balanced Salt Solution (HBSS) and incubated in Fluo-4/AM (1 μM) for 30 min in a 37°C incubator with 5% CO_2_. Cells were then centrifuged at 3000 rpm for 5 min and washed three times in HBSS. The green fluorescence intensity (516 nm) was quantified using a FACSAria flow cytometer (Becton Dickinson, Franklin Lakes, NJ, USA). For confocal imaging, cells were seeded on poly-l-lysine-coated glass cover slips 24 h after transfection with or without BAPTA/AM (50 μM for 1 h), as described above. Images were acquired with a confocal microscope.

### Subjects and plasma collection

Human plasma was prepared from fresh blood samples collected from PD patients or age-matched control subjects. A total of 50 patients diagnosed with PD (37 males and 13 females; mean age ± SD, 57.8 ± 10.7 years) according to UK PD Society Brain Bank criteria and 50 normal control subjects (41 males and 9 females; mean age ± SD, 62.3 ± 10.1) were included. Blood was collected in EDTA-coated tubes and separated by density gradient centrifugation at 400 ×*g* for 20 min. The plasma was stored at −80°C until use. Samples were randomized for analysis and were assayed at the same time on the same microtiter plate.

### Statistical analysis

For cell and animal experiments, protein expression levels were assessed by western blotting as previously reported [16]. Relative optical density values for α-Syn, myc, PP2A, and Src bands were normalized to that β-actin. The normalized ratio for the control group (untreated SK-N-SH or primary neurons) was taken as 100%. Phosphorylation levels of PP2A and Src are expressed as p-PP2A/PP2A and p-Src/Src ratios, respectively, with the ratio for the untreated group taken as 100%. Data for other groups are expressed as a percentage of the value for the control group. Results are presented as mean ± SD of at least three independent experiments. Differences between groups were evaluated by analysis of variance followed by the Bonferroni post hoc correction for multiple comparisons. Differences were considered significant at *P* < 0.05.

For clinical parameters, statistical analyses were performed using Prism 5 software (GraphPad Inc., La Jolla, CA, USA). Data are expressed as mean ± SD. The two-tailed independent samples t test was used to assess differences image and PP2A activity between PD patients and normal subjects. A Pearson correlation analysis was used to examine a possible association between PP2A activity and H&Y scores. *P* < 0.05 was considered statistically significant.

### Study approval

All animal studies were conducted in accordance with the National Institutes of Health (NIH) guidelines for humane treatment of animals (NIH Publication No. 80-23) and were approved by the Institutional Animal Care and Use Committee at Capital Medical University (approval no. AEEI-2014-120). Consecutive patients were recruited through the Outpatient Department at Xuanwu Hospitals, Capital Medical University, Beijing, China. All patients met the UK Brain Bank Criteria for idiopathic PD. Patients were tested during their “on” periods, while on their standard drug regimen. All participants provided written, informed consent for their participation in the study. The study protocol was approved by the Ethics Committee of Xuanwu Hospital (approval no. XW0020141017).
